# Associations of regular glucosamine use with all-cause and cause-specific mortality: a large prospective cohort study

**DOI:** 10.1136/annrheumdis-2020-217176

**Published:** 2020-04-06

**Authors:** Zhi-Hao Li, Xiang Gao, Vincent CH Chung, Wen-Fang Zhong, Qi Fu, Yue-Bin Lv, Zheng-He Wang, Dong Shen, Xi-Ru Zhang, Pei-Dong Zhang, Fu-Rong Li, Qing-Mei Huang, Qing Chen, Wei-Qi Song, Xian-Bo Wu, Xiao-Ming Shi, Virginia Byers Kraus, Xingfen Yang, Chen Mao

**Affiliations:** 1 Department of Epidemiology, School of Public Health, Southern Medical University, Guangzhou, China; 2 Department of Nutritional Sciences, The Pennsylvania State University, University Park, Pennsylvania, USA; 3 Jockey Club School of Public Health and Primary Care, The Chinese University of Hong Kong, Hong Kong, China; 4 National Institute of Environmental Health, Chinese Center for Disease Control and Prevention, Beijing, China; 5 Duke Molecular Physiology Institute and Division of Rheumatology, Department of Medicine, Duke University School of Medicine, Durham, North Carolina, USA; 6 Food Safety and Health Research Center, School of Public Health, Southern Medical University, Guangzhou, China

**Keywords:** epidemiology, cardiovascular disease, outcomes research

## Abstract

**Objectives:**

To evaluate the associations of regular glucosamine use with all-cause and cause-specific mortality in a large prospective cohort.

**Methods:**

This population-based prospective cohort study included 495 077 women and men (mean (SD) age, 56.6 (8.1) years) from the UK Biobank study. Participants were recruited from 2006 to 2010 and were followed up through 2018. We evaluated all-cause mortality and mortality due to cardiovascular disease (CVD), cancer, respiratory and digestive disease. HRs and 95% CIs for all-cause and cause-specific mortality were calculated using Cox proportional hazards models with adjustment for potential confounding variables.

**Results:**

At baseline, 19.1% of the participants reported regular use of glucosamine supplements. During a median follow-up of 8.9 years (IQR 8.3–9.7 years), 19 882 all-cause deaths were recorded, including 3802 CVD deaths, 8090 cancer deaths, 3380 respiratory disease deaths and 1061 digestive disease deaths. In multivariable adjusted analyses, the HRs associated with glucosamine use were 0.85 (95% CI 0.82 to 0.89) for all-cause mortality, 0.82 (95% CI 0.74 to 0.90) for CVD mortality, 0.94 (95% CI 0.88 to 0.99) for cancer mortality, 0.73 (95% CI 0.66 to 0.81) for respiratory mortality and 0.74 (95% CI 0.62 to 0.90) for digestive mortality. The inverse associations of glucosamine use with all-cause mortality seemed to be somewhat stronger among current than non-current smokers (p for interaction=0.00080).

**Conclusions:**

Regular glucosamine supplementation was associated with lower mortality due to all causes, cancer, CVD, respiratory and digestive diseases.

**Key messages**

**What is already known about this subject?**

- Although several epidemiological investigations indicated that glucosamine use might play a role in prevention of cancer, cardiovascular disease (CVD), and other diseases, only a few studies have evaluated the associations between glucosamine use and mortality outcomes, especially for cause-specific mortality.
- In addition, there is limited evidence on how potential modifiable factors affect associations of glucosamine use with all-cause and cause-specific mortality.

**What does this study add?**

- Regular glucosamine use is associated with a lower risk for total mortality (15%), CVD mortality (18%), cancer mortality (6%), respiratory mortality (27%) and digestive mortality (26%).
- Furthermore, the protective associations of glucosamine use against all-cause mortality appeared to be somewhat stronger in current smokers than non-current smokers.

**How might this impact on clinical practice or future developments?**

- These findings indicate that regular glucosamine use may provide a benefit against mortality among the general population; further clinical trials and possibly pharmacological studies may increase our understanding of any potential benefit of glucosamine supplement use.

## Background

Glucosamine is a non-vitamin, non-mineral specialty supplement commonly used to manage osteoarthritis and join pain.^1 2^ In most European countries, it is an approved prescription drug for osteoarthritis; however, in other countries such as the USA and Australia, it falls under the Dietary Supplements Health and Education Act and is one of the most popular supplements.^3 4^

Although the effectiveness of glucosamine supplementation for osteoarthritis and joint pain remains controversial,^1 5^ several human, animal and laboratory studies have suggested that glucosamine may have anti-inflammatory properties,^6–8^ which could decrease the risk of multiple diseases.^9–11^ In this context, several recent epidemiological investigations indicated that glucosamine use might play a role in prevention of cancer,^12–14^ cardiovascular disease (CVD)^15^ and other diseases.^16 17^ Nonetheless, only a few studies^15 18 19^ have evaluated the associations between glucosamine use and mortality outcomes, especially for cause-specific mortality. Moreover, there is only limited evidence on how potential modifiable factors affect associations of glucosamine use with all-cause and cause-specific mortality.^18^ For example, a recent study using data derived from the UK Biobank showed a weaker association of glucosamine use with CVD mortality in participants who were not current smokers compared with current smokers.^15^ However, the relatively small sample size in the previous analysis^18^ limited the ability to fully explore how associations with mortality due to all causes, cancer, respiratory and digestive disease vary with smoking status.

In this large-scale prospective cohort study of nearly half a million UK adults, we evaluated the association between regular glucosamine supplement use and mortality from all causes, CVD, cancer, respiratory disease and digestive disease. Furthermore, we analysed the potential effect modification by several other risk factors for all-cause and cause-specific mortality.

## Methods

### Study population

The UK Biobank is a very large, population-based prospective cohort study designed to improve the prevention, diagnosis and treatment of a wide range of diseases and to promote health throughout society.^20^ Details of the study design and population have been reported elsewhere.^20^ In brief, the UK Biobank recruited over 500 000 men and women aged 40–70 years between 2006 and 2010 from across the UK. Participants provided detailed self-reported data via a touch screen questionnaire and a verbal interview with trained nurses at the assessment centres at baseline, and a wide range of physical measurements were collected.

### Exposure assessment

Participants attended 1 of 22 assessment centres across the UK where they completed a touch screen questionnaire. One of the questions asked, ‘Do you regularly take any of the following?’, and participants could select answers from a list of supplements that included glucosamine. From this information, we defined the regular use of glucosamine as ‘1=yes’ and ‘0=no’.

### Ascertainment of deaths

The UK Biobank undertook comprehensive data linkage for mortality status. Information about date and cause of death was obtained from the Information Centre (for England and Wales) and the National Health Service Central Register Scotland (for Scotland).^20^ Further detailed information about the linkage procedure is available at http://content.digital.nhs.uk/services. The International Classification of Diseases, 10th Revision (ICD-10) codes were used to identify the causes of death. For this analysis, we measured all-cause mortality and mortality due to CVD (codes I00-I99), cancer (codes C00-C97), respiratory diseases (codes J09-J98) and digestive diseases (codes K20-K93). The participants were followed up from the date of recruitment (between 2006 and 2010) to the date of death or the end of follow-up (14 February 2018 for England and Wales and 1 January 2017 for Scotland), whichever occurred first.

## Covariates

The UK Biobank used a baseline touch screen questionnaire to assess several potential confounders: sociodemographic characteristics (age, sex, ethnicity, Townsend Deprivation Index, education and average total annual household income), lifestyle behaviours (smoking status, alcohol consumption, physical activity, body mass index (BMI) and vegetable and fruit consumption), health conditions (CVD (myocardial infarction, angina or stroke), respiratory disease (chronic obstructive pulmonary disease or emphysema), cancer, digestive disease (liver failure, cirrhosis or alcoholic liver disease), dementia, depression, longstanding illness, hypertension, diabetes and high cholesterol), drug use (chondroitin, aspirin and other non-steroidal anti-inflammatory drug (NSAID) use), vitamin supplementation (vitamin A, vitamin B, vitamin C, vitamin D, vitamin E, multivitamin and folic acid) and mineral and other dietary supplementation (calcium, iron, zinc, selenium and fish oil)). Education was coded as degree (college/university degree), or no degree, which was derived from the questionnaire. BMI was calculated by dividing a participant’s weight by the square of his or her height in metres (kg/m^2^). The Townsend Deprivation Index is a composite measure of deprivation based on non-home ownership, non-car ownership, unemployment and household overcrowding,^21^ which represents the participant’s socioeconomic status. According to WHO recommendations on physical activity for health,^22^ we categorised participants as <150 or ≥150 min/week, which was based on total moderate physical activity minutes per week. Hypertension was defined as a self-reported history of hypertension, a systolic blood pressure ≥140 mm Hg, a diastolic blood pressure ≥90 mm Hg or the report of antihypertensive drugs use. Arthritis defined by ICD-10 codes was obtained from hospital records, while the information on the other conditions was obtained by self-report with augmentation of these data using ICD-10 from hospital records. Further details of these measurements are available on the UK Biobank website (www.ukbiobank.ac.uk).

### Statistical analysis

Baseline characteristics are presented as the mean (SD) for continuous variables and number (%) for categorical variables. To minimise the potential for inferential bias, we conducted multiple imputation with chained equations to deal with missing values,^23 24^ and five datasets were imputed. All variables used in the analyses were included in the imputation model. The interaction was included with a product of the two variables (ie, glucosamine use and smoking status) and computed after imputation in the imputed file. Due to low proportions of missing data, we regard this way to handle interactions to work satisfactorily. Detailed information on the number of missing variables is shown in online supplementary table S1.

10.1136/annrheumdis-2020-217176.supp1 Supplementary data

Cox proportional hazards models were performed to calculate HRs and 95% CIs for associations of glucosamine use with risk of all-cause and cause-specific mortality. The models for mortality from CVD, respiratory disease, cancer or digestive disease excluded participants with a history of CVD, respiratory disease, cancer or digestive disease at baseline, respectively. For the analyses, we ran two models: the basic model (model 1) was adjusted for age and sex (women or men), and the fully adjusted model (model 2) was adjusted for the same factors as model 1 and included ethnicity (white or others), education (degree or no degree), household income (<£18 000, £18 000–£30 999, £31 000–£51 999, £52 000–£100 000 and >£100 000), Townsend Deprivation Index, BMI, smoking status (current, former or never), alcohol consumption (current, former or never), physical activity (<150 or ≥150 min/week), vegetable consumption (<2.0, 2.0–3.9 or ≥4.0 servings/day), fruit consumption (<2.0, 2.0–3.9 or ≥4.0 servings/day), diabetes (yes or no), hypertension (yes or no), high cholesterol (yes or no), CVD (yes or no), cancer (yes or no), respiratory disease (yes or no), digestive disease (yes or no), dementia (yes or no), depression (yes or no), longstanding illness (yes or no), arthritis (yes or no), statin use (yes or no), chondroitin use (yes or no), aspirin use (yes or no), non-aspirin NSAID use (yes or no), vitamin supplement use (yes or no; multivitamin, folic acid, vitamin A, vitamin B, vitamin C, vitamin D or vitamin E) and mineral and other dietary supplement use (yes or no; calcium, iron, zinc, selenium or fish oil). The proportional hazards assumption was tested using a Schoenfeld residuals plot,^25^ and we found no violation of the assumption in this study. In addition to conventional multivariate Cox regression analysis, we constructed a propensity score for adjustment; we obtained the propensity score using a logistic regression that included the aforementioned baseline covariates for glucosamine use.

We conducted a stratified analysis to assess potential modification effects by the following factors: sex (women or men), age (<60 or ≥60 years), ethnicity (white or others), obesity (yes or no), physical activity (<150 or ≥150 min/week), current smoking status (yes or no), current alcohol consumption status (yes or no), hypertension (yes or no), diabetes (yes or no), high cholesterol (yes or no), statin use (yes or no) and aspirin use (yes or no). We evaluated potential effect modification by modelling the cross-product term of the stratifying variable with glucosamine use in a fully adjusted model.

We also conducted a series of sensitivity analyses to test the robustness of our results. First, because participants who took glucosamine also tended to take other supplements, or chondroitin more often than participants who did not take glucosamine, we performed sensitivity analyses by excluding participants who used any other supplements and chondroitin, respectively. Second, we excluded participants who died within 2 years of follow-up to minimise potential reverse causation. Third, we removed participants with missing values for covariates. We used R V.3.6.1 (R Development Core Team, Vienna, Austria) for all statistical analyses, and p<0.05 (two-sided) were considered significant. Because we tested multiple interactions, we conservatively corrected for multiple testing using Bonferroni correction and set significance level of 0.05/60=0.00083.

## Results

### Baseline characteristics

**Figure 1:**
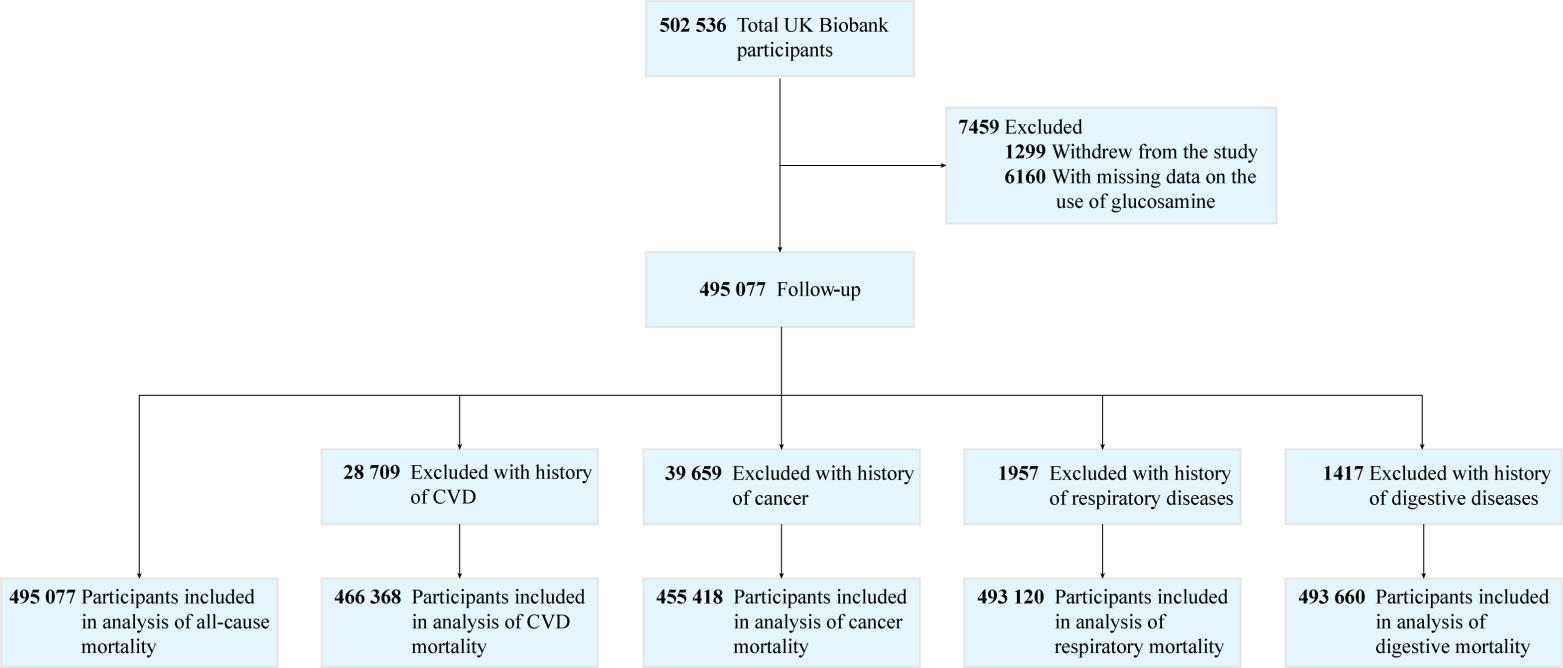
Flow chart of participant enrolment. Cardiovascular disease (CVD) (myocardial infarction, angina or stroke), respiratory disease (chronic obstructive pulmonary disease or emphysema) and digestive disease (liver failure, cirrhosis or alcoholic liver disease) at baseline.

We excluded participants who withdrew from the study (1299), and those with missing data on the use of glucosamine (6160). Our final analysis included 495 077 participants with data for all-cause mortality. Participants with relevantly prevalent disease were excluded per cause-specific mortality outcome, leaving 466 368 participants for the analyses of CVD mortality, 455 418 for cancer mortality, 493 120 for respiratory mortality and 493 660 for digestive mortality (figure 1).

Of the 495 077 participants (mean (SD) age, 56.6 (8.1) years), 269 549 (54.4%) were women. Overall, 94 346 (19.1%) participants reported glucosamine use at baseline. Compared with non-users, glucosamine users were older, more likely to be women, current non-smokers, more physically active and with higher comorbidities, including cancer, hypertension and arthritis, but a lower prevalence of CVD and diabetes (table 1). Glucosamine users also tended to take more chondroitin, non-aspirin NSAIDs, vitamins and minerals and other dietary supplements than nonusers.

**Table 1:** Baseline characteristics of study participants by glucosamine use

Characteristics	Overall (n=495 077)	Glucosamine non-users (n=400 731)	Glucosamine users (n=94 346)
Age, mean (SD), years	56.55 (8.09)	55.95 (8.20)	59.08 (7.07)
Women	269 549 (54.4)	210 497 (52.5)	59 052 (62.6)
TDI, mean (SD)	−1.31 (3.09)	−1.20 (3.14)	−1.79 (2.79)
Education			
Degree	160 288 (32.4)	129 146 (32.2)	31 142 (33.0)
No degree	334 789 (67.6)	271 585 (67.8)	63 204 (67.0)
Ethnicity			
White	455 861 (92.1)	367 313 (91.7)	88 548 (93.9)
Others	39 216 (7.9)	33 418 (8.3)	5798 (6.1)
Household income (£)			
<18 000	116 815 (23.6)	95 680 (23.9)	21 135 (22.4)
18 000–30 999	127 517 (25.8)	100 419 (25.1)	27 098 (28.7)
31 000–51 999	127 427 (25.7)	102 879 (25.7)	24 548 (26.0)
52 000–100 000	97 565 (19.7)	80 314 (20.0)	17 251 (18.3)
>100 000	25 753 (5.2)	21 439 (5.3)	4314 (4.6)
BMI, mean (SD), kg/m^2^	27.43 (4.80)	27.45 (4.84)	27.36 (4.65)
Smoking status			
Never	271 144 (54.8)	219 107 (54.7)	52 037 (55.2)
Former	171 668 (34.7)	135 486 (33.8)	36 182 (38.4)
Current	52 265 (10.6)	46 138 (11.5)	6127 (6.5)
Alcohol consumption			
Never	21 931 (4.4)	18 688 (4.7)	3243 (3.4)
Former	17 858 (3.6)	15 136 (3.8)	2722 (2.9)
Current	455 288 (92.0)	366 907 (91.6)	88 381 (93.7)
Physical activity (min/week)			
<150	228 019 (46.1)	189 753 (47.4)	38 266 (40.6)
≥150	267 058 (53.9)	210 978 (52.6)	56 080 (59.4)
Vegetable consumption (servings/day)			
<2.0	97 853 (19.8)	83 776 (20.9)	14 077 (14.9)
2.0–3.9	222 743 (45.0)	179 783 (44.9)	42 960 (45.5)
≥4.0	174 481 (35.2)	137 172 (34.2)	37 309 (39.5)
Fruit consumption (servings/day)			
<2.0	136 458 (27.6)	118 612 (29.6)	17 846 (18.9)
2.0–3.9	201 446 (40.7)	163 136 (40.7)	38 310 (40.6)
≥4.0	157 173 (31.7)	118 983 (29.7)	38 190 (40.5)
Supplement or drug use			
Vitamin	157 133 (31.7)	104 719 (26.1)	52 414 (55.6)
Minerals and other dietary supplements	184 377 (37.2)	118 971 (29.7)	65 406 (69.3)
Aspirin	66 052 (13.3)	53 402 (13.3)	12 650 (13.4)
Statin	56 544 (11.4)	46 186 (11.5)	10 358 (11.0)
Non-aspirin NSAIDs	71 109 (14.4)	53 152 (13.3)	17 957 (19.0)
Chondroitin	7813 (1.6)	1581 (0.4)	6232 (6.6)
Health conditions			
CVD	28 709 (5.8)	24 621 (6.1)	4088 (4.3)
Cancer	39 659 (8.0)	31 506 (7.9)	8153 (8.6)
Diabetes	25 968 (5.2)	22 517 (5.6)	3451 (3.7)
Hypertension	279 956 (56.5)	225 247 (56.2)	54 709 (58.0)
Respiratory diseases	1957 (0.4)	1555 (0.4)	402 (0.4)
Digestive diseases	1417 (0.3)	1259 (0.3)	158 (0.2)
High cholesterol	86 406 (17.5)	70 408 (17.6)	15 998 (17.0)
Arthritis	23 217 (4.7)	15 440 (3.9)	7777 (8.2)
Dementia	219 (0.0)	184 (0.0)	35 (0.0)
Depression	76 642 (15.5)	53 848 (13.4)	15 133 (16.0)
Longstanding illness	162 123 (32.7)	131 024 (32.7)	31 099 (33.0)

Values are numbers (%) unless stated otherwise.

BMI, body mass index; CVD, cardiovascular disease; NSAID, non-steroidal anti-inflammatory drug; TDI, Townsend Deprivation Index.

### Associations of glucosamine use with all-cause and cause-specific mortality

During a median follow-up of 8.9 years (IQR 8.3–9.7 years), we documented 19 882 all-cause deaths, 3802 CVD deaths, 8090 cancer deaths, 3380 respiratory deaths and 1061 digestive disease deaths. In age-adjusted and sex-adjusted analyses, we found significant inverse associations between glucosamine use and risk of mortality due to all-cause, CVD, cancer, respiratory disease and digestive disease (all p<0.001) (table 2). In the multivariable adjusted analyses, the HRs associated with glucosamine use were 0.85 (95% CI 0.82 to 0.89) for all-cause mortality; 0.82 (95% CI 0.74 to 0.90) for CVD mortality; 0.94 (95% CI 0.88 to 0.99; p=0.031) for cancer mortality; 0.73 (95% CI 0.66 to 0.81) for respiratory disease mortality and 0.74 (95% CI 0.62 to 0.90) for digestive disease mortality (table 2).

**Table 2:** Associations of glucosamine supplement use with risk of all-cause and cause-specific mortality

Outcomes	Glucosamine non-users	Glucosamine users	Model 1*		Model 2†		Propensity score adjusted	
			HR (95% CI)	P value	HR (95% CI)	P value	HR (95% CI)	P value
All-cause mortality	16 665 (4.2)	3217 (3.4)	0.70 (0.67 to 0.73)	<0.001	0.85 (0.82 to 0.89)	<0.001	0.82 (0.79 to 0.86)	<0.001
CVD mortality	3202 (0.8)	600 (0.7)	0.67 (0.61 to 0.73)	<0.001	0.82 (0.74 to 0.90)	<0.001	0.78 (0.71 to 0.86)	<0.001
Cancer mortality	6571 (2.2)	1519 (2.1)	0.82 (0.78 to 0.87)	<0.001	0.94 (0.88 to 0.99)	0.031	0.90 (0.85 to 0.96)	0.001
Respiratory disease mortality	2917 (0.7)	463 (0.5)	0.56 (0.51 to 0.62)	<0.001	0.73 (0.66 to 0.81)	<0.001	0.68 (0.61 to 0.76)	<0.001
Digestive disease mortality	914 (0.2)	147 (0.2)	0.61 (0.51 to 0.73)	<0.001	0.74 (0.62 to 0.90)	<0.001	0.73 (0.60 to 0.88)	<0.001

Values are numbers (%) unless stated otherwise.

*Model 1: adjusted for age and sex.

†Model 2: additionally adjusted for Townsend Deprivation Index, ethnicity, education, household income, body mass index, fruit consumption, vegetable consumption, smoking status, alcohol consumption, physical activity, diabetes, hypertension, high cholesterol, CVD, cancer, respiratory disease, digestive disease, arthritis, dementia, depression, longstanding illness, statin use, chondroitin use, aspirin use, non-aspirin NSAID use, vitamin supplementation and mineral and other dietary supplementation.

CVD, cardiovascular disease; NSAID, non-steroidal anti-inflammatory drug.

### Subgroup analyses

**Figure 2:**
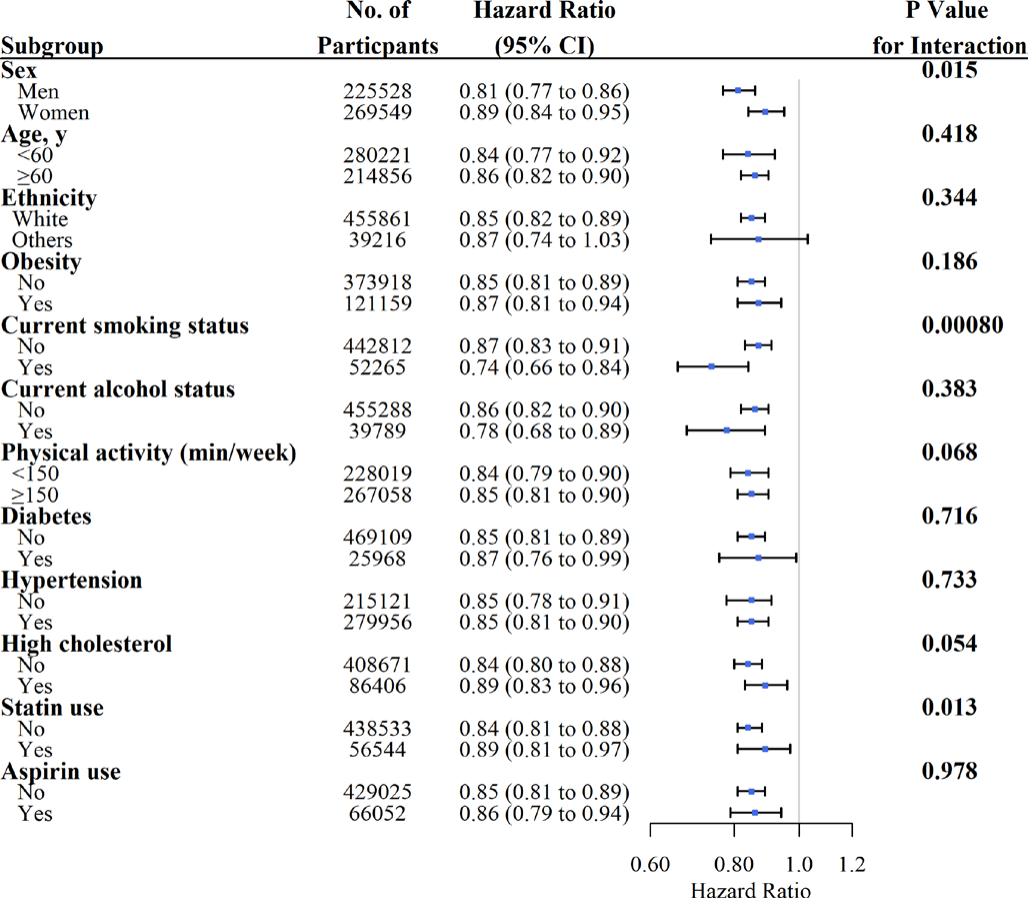
Association of glucosamine supplement use and risk of all-cause mortality stratified by potential risk factors. Results were adjusted for age, sex, Townsend Deprivation Index, ethnicity, education, household income, body mass index, fruit consumption, vegetable consumption, smoking status, alcohol consumption, physical activity, diabetes, hypertension, high cholesterol, cardiovascular disease, cancer, respiratory disease, digestive disease, arthritis, dementia, depression, longstanding illness, statin use, chondroitin use, aspirin use, non-aspirin non-steroidal anti-inflammatory drug use, vitamin supplementation and mineral and other dietary supplementation.

**Figure 3:**
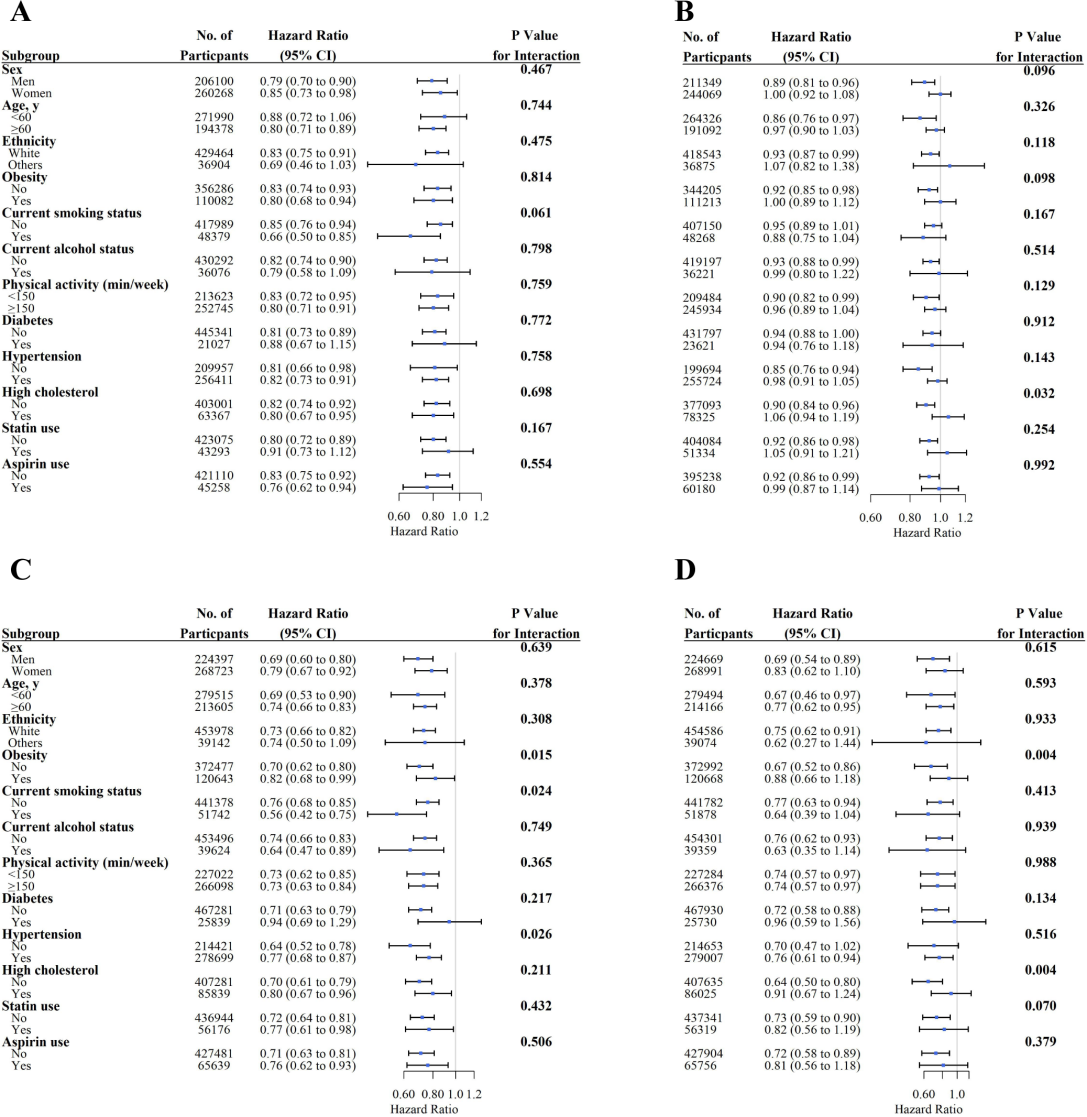
Associations of glucosamine supplement use and risk of cause-specific mortality stratified by potential risk factors. (A) Cardiovascular disease (CVD) mortality; (B) cancer mortality; (C) respiratory disease mortality; (D) digestive disease mortality. Results were adjusted for age, sex, Townsend Deprivation Index, ethnicity, education, household income, body mass index, fruit consumption, vegetable consumption, smoking status, alcohol consumption, physical activity, diabetes, hypertension, high cholesterol, CVD, cancer, respiratory disease, digestive disease, arthritis, dementia, depression, longstanding illness, statin use, chondroitin use, aspirin use, non-aspirin non-steroidal anti-inflammatory drug use, vitamin supplementation and mineral and other dietary supplementation.

We conducted stratified analyses for associations of glucosamine use with all-cause and cause-specific mortality according to potential risk factors using the fully adjusted model (figures 2 and 3). We observed a significant interaction effect between glucosamine use and current smoking on the risks of all-cause mortality (p for interaction=0.00080). However, the associations between glucosamine use and all-cause and cause-specific mortality were not significantly modified by sex, age, ethnicity, obesity, current alcohol status, physical activity, diabetes, statin and aspirin use (figures 2 and 3).

### Sensitivity analyses

The associations of glucosamine use with all-cause and cause-specific mortality did not change appreciably when we excluded participants who died within 2 years of follow-up (see online supplementary table S2); when we excluded participants who used any other supplements (see online supplementary table S3); nor when we excluded participants with missing values for covariates (see online supplementary table S4). Likewise, when the analyses of glucosamine were restricted to non-users of chondroitin, material changes in the results were not observed (see online supplementary table S5).

## Discussion

In this large population-based cohort study involving 495 077 individuals, we found that regular glucosamine use was significantly associated with a 15% lower risk of total mortality and 18% for CVD mortality; 6% for cancer mortality; 27% for respiratory mortality and 26% for digestive mortality. These associations were independent of other potential confounders, including sociodemographic factors, lifestyle behaviours, health status, drug use and other supplements use. Furthermore, the protective associations of glucosamine use against all-cause mortality appeared to be somewhat stronger in current smokers than non-current smokers.

Our results showed that glucosamine use was reported by 19.1% of participants; similarly, glucosamine use was reported by 22.0% of the Australian population aged 45+ years.^4^ Our results are consistent with findings from several previous studies, which indicated an inverse association between glucosamine use and mortality. In the Vitamins and Lifestyle (VITAL) study, glucosamine use was significantly associated with a lower risk of all-cause mortality.^18^ An animal study found that glucosamine may prolong life span by mimicking a low carbohydrate diet,^26^ while the protective effect of a low carbohydrate diet on mortality has been demonstrated in population-based studies.^27 28^ Moreover, these observations echoes the results of a study using the UK Biobank data with a mean of 7 years of follow-up, of which Ma *et al* 15 reported that habitual glucosamine use was associated with a lower risk of CVD mortality. Our current study, with nearly two additional years of follow-up, has provided further evidence supporting the association between glucosamine use and a lower risk of CVD mortality. Besides, our study has shown a reduction of cancer-specific mortality in association with glucosamine use agrees with the VITAL study, which found an inverse association between glucosamine use and the risk of colorectal^12^ and lung cancer.^29^ With regard to respiratory-specific mortality, only one study supports our finding that the use of glucosamine is associated with a reduced risk of death from respiratory disease.^18^ From a mechanistic perspective, our observation is supported by the fact that anti-inflammatory drugs have been proposed as an approach to hinder the progress of chronic obstructive pulmonary disease.^30^

To our knowledge, the association between glucosamine use and digestive disease mortality has not been reported previously. Although low dose aspirin reduces the risk of CVD events, it may increase the risk of upper gastrointestinal complications, particularly when it is administered in conjunction with NSAIDs or even acetaminophen.^31 32^ Unlike aspirin or NSAIDs, glucosamine is considered relatively safe.^33^ Future studies are needed to investigate the associations of glucosamine use with digestive diseases and mortality.

Glucosamine and chondroitin supplements are often taken together in a single daily supplements,^1^ and it is therefore possible that our observed associations are driven by either of these supplements. To address this issue, we performed sensitivity analyses examining the associations of glucosamine use alone (excluding participants who took chondroitin) with all-cause and cause-specific mortality. We found that the estimates did not change substantially. Therefore, it is likely that glucosamine use may reduce the risk of mortality, regardless of the co-administration of chondroitin.

Several potential mechanisms could explain the inverse association between glucosamine use and mortality. First, nuclear factor-κB (NF-κB) has been implicated in several diseases, such as inflammation-related CVD and cancers.^34^ Glucosamine use may affect inflammation by inhibiting the transcription factor NF-κB from translocating to the nucleus,^6 35^ reducing inflammation and thus lowering related mortality. Indeed, several previous studies have demonstrated that such anti-inflammatory properties of glucosamine might promote healthy outcomes.^6–8^ For example, glucosamine use was found to be associated with a significant reduction of concentrations of C reactive protein.^8^ Reduction of this important marker for systemic inflammation^36^ could be related to subsequent lowering of morbidity and mortality risk.^37 38^ Moreover, orally administered glucosamine reduced the markers of inflammation in peripheral blood, as well as atherosclerotic-induced femoral lesions, in a combined rabbit model of chronic arthritis and atherosclerosis.^39^ Glucosamine also prevented the development of inflammation-associated aortic lesions.^39^ Aside from reducing inflammation, an animal study reported that glucosamine use could trigger a mimic response of a low carbohydrate diet, via reducing glycolysis and increasing amino acid catabolism in mice.^26^ This could explain the linkage between glucosamine use and its protective effect, as population-based studies found that low carbohydrate diets are indeed related to a reduced risk of mortality.^27 40^ In addition, several trials reported that a low carbohydrate diet could promote beneficial health outcomes.^41 42^ Mechanisms other than anti-inflammation, reducing glycolysis and increasing amino acid catabolism might also be involved in mediating our observed outcomes. Future studies are needed to explore the diverse pharmacological roles of glucosamine on different health outcomes.

We observed that the association between glucosamine use and all-cause mortality varied by smoking status, with a significant inverse association observed among those who are current smokers. One of the possible explanation on why we observe a stronger effect among current smokers is that, as they are at a state of higher inflammatory stress at baseline, the anti-inflammatory actions of glucosamine may offer stronger benefit.^43^ It would be interesting to clarify how current smokers could benefit from glucosamine use, particular on reducing smoking-related mortality.

### Strengths and limitations

Our study has several major strengths, including the large sample size, the prospective population-based cohort study design and minimal loss to follow-up. It is noteworthy that the wealth of information on socioeconomic factors, lifestyle, diseases history and other covariates of the UK Biobank dataset has enabled us to perform comprehensive sensitivity and subgroup analyses. Nevertheless, our study also has some limitations. First, while data collection on dietary supplements intake was not conducted in a clinical setting in order to encourage more truthful reporting, the UK Biobank did not gather detailed information on the dosage, forms or duration of glucosamine use. Second, as the time of taking glucosamine was unknown in the UK Biobank, deaths that occurred during the exposure but before the follow-up date were not able to be counted. This may exaggerate the estimated protective effect of taking glucosamine on the basis of what is called an ‘immortal time bias’. Third, regular glucosamine use may be a marker for a healthy lifestyle, but it is hard to distinguish the confounding effects of a healthy lifestyle from the impact of regular supplementations in an observational study. Although we had carefully adjusted for potential confounding lifestyle-related factors in our analyses, we could not exclude the possibility that the results were confounded by unmeasured lifestyle-related factors. In general, with the current observational study design the possibility of residual confounding due to imprecise measurements or unknown factors cannot be excluded for all findings in our study, despite our careful adjustment of all measured confounders. Fourth, in general, 20–100 imputed datasets are recommended,^24^ while in this study 5 datasets were imputed. Due to low proportions of missing data, we regard five imputed datasets to work satisfactorily. Lastly, although the UK Biobank represents a large and unique resource, low response rate (5.5%) could lead to selection biases, potentially limiting generalisability of the results in the wider UK population.^20^

## Conclusions

In summary, the results of this large-scale prospective cohort study show that a considerable proportion (19.1%) of the UK population reported regular use of glucosamine supplements. We observed that regular use of glucosamine supplements is associated with lower mortality due to all causes, CVD, cancer, respiratory disease and digestive disease. Further clinical trials and possibly pharmacological studies may increase our understanding of any potential benefit of glucosamine supplement use.
