# Supplementary material for: Associations of regular glucosamine use with all-cause and cause-specific mortality: a large prospective cohort study
Source: Ann Rheum Dis. 2020 Apr 6;79(6):829–36. doi: 10.1136/annrheumdis-2020-217176 (PMC7286049; doi:10.1136/annrheumdis-2020-217176)
Supplement: Supplementary data [file annrheumdis-2020-217176supp001.pdf]

Supplement Tables

Table S1. The numbers (percentage) of the missing variables

Characteristics[*]	Number (%) with missing data
TDI, mean (SD)	616 (0.1)
Household income (£)	
<18 000	20 222 (17.3)
18 000 to 30 999	19 751 (15.5)
31 000 to 51 999	17 037 (13.4)
52 000 to 100 000	11 660 (11.9)
>100 000	2964 (11.5)
BMI, mean (SD), kg/m^2	2552 (0.5)
Smoking status	
Never	955 (0.4)
Former	688 (0.4)
Current	219 (0.4)
Alcohol consumption	
Never	27 (0.1)
Former	15 (0.1)
Current	525 (0.1)
Physical activity (min/week)	
<150	4984 (2.1)
≥150	5468 (2.0)
Vegetable consumption (servings/day)	
<2.0	2272 (2.3)
2.0-3.9	4010 (1.8)
≥4.0	2906 (1.7)
Fruit consumption (servings/day)	
<2.0	2492 (1.8)
2.0-3.9	2367 (1.2)
≥4.0	1887 (1.2)

*List of only the variables with missing data; BMI: body mass index; SD: standard deviation; TDI: Townsend Deprivation Index.

Li Z-H, *et al. Ann Rheum Dis* 2020; 79:829–836. doi: 10.1136/annrheumdis-2020-217176

Table S2. Associations of Glucosamine Supplement Use with the Risk of All-Cause and Cause-Specific Mortality after Excluding Participants who Experienced an Outcome Event during the First Two Years of Follow-up.

Outcomes	Glucosamine nonusers	Glucosamine users	Model 2*	
			HR (95% CI)	*P* value
All-cause mortality	14 512 (3.6)	2905 (3.1)	0.87 (0.83-0.91)	<0.001
CVD mortality	2793 (0.8)	536 (0.6)	0.82 (0.74-0.91)	<0.001
Cancer mortality	5954 (1.6)	1425 (1.6)	0.96 (0.90-1.02)	0.207
Respiratory disease mortality	2593 (0.6)	436 (0.5)	0.76 (0.69-0.85)	<0.001
Digestive disease mortality	792 (0.2)	134 (0.1)	0.77 (0.63-0.94)	0.008

Values are numbers (%) unless stated otherwise;

HR: hazard ratio; CI: confidence interval; CVD: cardiovascular disease;

*Model 2: adjusted for age, sex, Townsend Deprivation Index, ethnicity, education, household income, body mass index, fruit consumption, vegetable consumption, smoking status, alcohol consumption, physical activity, diabetes, hypertension, high cholesterol, CVD, cancer, respiratory disease, digestive disease, arthritis, dementia, depression, longstanding illness, statin use, chondroitin use, aspirin use, non-aspirin NSAID use, vitamin supplementation, and mineral and other dietary supplementation.

Li Z-H, *et al. Ann Rheum Dis* 2020; 79:829–836. doi: 10.1136/annrheumdis-2020-217176

Table S3. Associations of Glucosamine Supplement Use with the Risk of All-Cause and Cause-Specific Mortality after Excluding Participants who Took Vitamins and Mineral Supplements.

Outcomes	Glucosamine nonusers	Glucosamine users	Model 2*	
			HR (95% CI)	*P* value
All-cause mortality	9736 (4.0)	590 (3.4)	0.83 (0.77-0.91)	<0.001
CVD mortality	1885 (0.8)	112 (0.7)	0.82 (0.67-0.99)	0.042
Cancer mortality	3991 (1.8)	290 (1.8)	0.93 (0.82-1.05)	0.223
Respiratory disease mortality	1627 (0.7)	79 (0.5)	0.70 (0.56-0.89)	0.002
Digestive disease mortality	508 (0.2)	26 (0.1)	0.78 (0.52-1.17)	0.226

Values are numbers (%) unless stated otherwise;

HR: hazard ratio; CI: confidence interval; CVD: cardiovascular disease;
*Model 2: adjusted for age, sex, Townsend Deprivation Index, ethnicity, education, household income, body mass index, fruit consumption, vegetable consumption, smoking status, alcohol consumption, physical activity, diabetes, hypertension, high cholesterol, CVD, cancer, respiratory disease, digestive disease, arthritis, dementia, depression, longstanding illness, statin use, chondroitin use, aspirin use, and non-aspirin NSAID use.

Li Z-H, *et al. Ann Rheum Dis* 2020; 79:829–836. doi: 10.1136/annrheumdis-2020-217176

Table S4. Associations of Glucosamine Supplement Use with the Risk of All-Cause and Cause-Specific Mortality after Excluding Participants with Missing Covariate Data.

Outcomes	Glucosamine nonusers	Glucosamine users	Model 2*	
			HR (95% CI)	*P* value
All-cause mortality	16 665 (4.2)	3217 (3.4)	0.86 (0.82-0.91)	<0.001
CVD mortality	3202 (0.8)	600 (0.7)	0.81 (0.72-0.90)	<0.001
Cancer mortality	6571 (1.8)	1519 (1.8)	0.94 (0.88-1.01)	0.071
Respiratory disease mortality	2917 (0.7)	463 (0.5)	0.78 (0.69-0.88)	<0.001
Digestive disease mortality	914 (0.2)	147 (0.2)	0.75 (0.60-0.93)	0.008

Values are numbers (%) unless stated otherwise;

HR: hazard ratio; CI: confidence interval; CVD: cardiovascular disease;

*Model 2: adjusted for age, sex, Townsend Deprivation Index, ethnicity, education, household income, body mass index, fruit consumption, vegetable consumption, smoking status, alcohol consumption, physical activity, diabetes, hypertension, high cholesterol, CVD, cancer, respiratory disease, digestive disease, arthritis, dementia, depression, longstanding illness, statin use, chondroitin use, aspirin use, non-aspirin NSAID use, vitamin supplementation, and mineral and other dietary supplementation.

Li Z-H, *et al. Ann Rheum Dis* 2020; 79:829–836. doi: 10.1136/annrheumdis-2020-217176

Table S5. Associations of Glucosamine Supplement Use with the Risk of All-Cause and Cause-Specific Mortality after Excluding Participants who Took Chondroitin.

Outcomes	Glucosamine nonusers	Glucosamine users	Model 2*	
			HR (95% CI)	*P* value
All-cause mortality	16 568 (4.2)	3015 (3.4)	0.85 (0.82-0.89)	<0.001
CVD mortality	3184 (0.9)	563 (0.7)	0.82 (0.74-0.90)	<0.001
Cancer mortality	6539 (1.8)	1419 (1.8)	0.94 (0.88-0.99)	0.032
Respiratory disease mortality	2899 (0.7)	439 (0.5)	0.73 (0.66-0.82)	<0.001
Digestive disease mortality	909 (0.2)	135 (0.1)	0.74 (0.61-0.89)	0.001

Values are numbers (%) unless stated otherwise;

HR: hazard ratio; CI: confidence interval; CVD: cardiovascular disease;

*Model 2: adjusted for age, sex, Townsend Deprivation Index, ethnicity, education, household income, body mass index, fruit consumption, vegetable consumption, smoking status, alcohol consumption, physical activity, diabetes, hypertension, high cholesterol, CVD, cancer, respiratory disease, digestive disease, arthritis, dementia, depression, longstanding illness, statin use, chondroitin use, aspirin use, non-aspirin NSAID use, vitamin supplementation, and mineral and other dietary supplementation.

Li Z-H, *et al. Ann Rheum Dis* 2020; 79:829–836. doi: 10.1136/annrheumdis-2020-217176
